# More trees, more naturalness, and greater willingness to engage in physical activity: an image-based study

**DOI:** 10.3389/fpubh.2026.1881655

**Published:** 2026-06-17

**Authors:** Yanxia Zhao, Li Huang, Haodong Tian, Yunfei Tao, Zhiyuan Tan, Hengzhi Deng, Zhengyang Mei, Lunxin Chen, Xing Zhang, Hansen Li

**Affiliations:** 1 College of Physical Education, Chongqing University, Chongqing, China; 2 Faculty of Psychology, Southwest University, Chongqing, China; 3 College of Physical Education, Southwest University, Chongqing, China; 4 School of Sport Training, Chengdu Sport University, Chengdu, Sichuan, China; 5 Faculty of Sport and Physical Education, University of Belgrade, Belgrade, Serbia; 6 Faculty of Sports and Exercise Science, University of Malaya, Kuala Lumpur, Malaysia; 7 School of Physical Education and Sports, Central China Normal University, Wuhan, China; 8 Department of Physical Education and Sport, Faculty of Sport Sciences, University of Granada, Granada, Spain; 9 School of Physical Education, Sichuan Agricultural University, Ya’an, China

**Keywords:** active living, city, green exercise, health, nature, physical activity

## Abstract

**Introduction:**

Urban green spaces may promote health partly by encouraging physical activity, but it remains unclear which visible vegetation elements most strongly shape perceived naturalness and activity-related responses. This image-based study examined whether visible vegetation, particularly visible tree proportion, was associated with perceived naturalness and willingness to engage in physical activity in urban environments.

**Methods:**

An online image-based questionnaire was conducted among 337 university students in Ya’an, China. Participants evaluated 40 photographs of typical urban streets and open spaces in Yucheng District. A DeepLabv3 semantic segmentation model pretrained on the ADE20K dataset was used to quantify the pixel proportions of three vegetation-related labels: Tree, Grass, and Other plant. Participants rated perceived naturalness on a 4-point scale and willingness to engage in physical activity on an 11-point scale. Analyses included image-level Pearson correlations, generalized estimating equations, receiver operating characteristic analysis, mediation analysis, and image-level sensitivity analyses.

**Results:**

Perceived naturalness was strongly correlated with physical activity willingness (*r* = 0.949, *p* < 0.001). Tree, Grass, and Other plant proportions were positively associated with perceived naturalness, whereas only Tree and Other plant showed significant positive associations with physical activity willingness in the main GEE models. Tree proportion showed the most consistent pattern of associations across the main and sensitivity analyses. ROC analysis indicated that Tree proportion had good ability to identify spaces perceived as natural (AUC = 0.90), with an exploratory optimal cut-off of 27.7%. Mediation analysis further showed that perceived naturalness significantly mediated the associations of Tree and Other plant proportions with physical activity willingness.

**Discussion:**

Visible vegetation, especially visible tree proportion, may contribute to stronger perceived naturalness and greater willingness to be physically active in urban spaces. These findings highlight the potential importance of visible trees in urban greening and active-living design. However, because this study used static images and measured willingness rather than actual behavior, the results should be interpreted as preliminary evidence concerning environmental perception and activity tendency.

## 1 Introduction

Natural environments are the settings on which humans historically depended for survival and remain crucial for maintaining health in modern society, particularly in the context of ongoing global urbanization (1, 2). A large body of research has examined the health benefits of spaces dominated by plants, water, and other natural elements (3–6). Among these, plant-dominated green spaces have received the greatest attention. Green space generally refers to land covered by vegetation (7). In the literature, this often includes vegetated areas in urban parks, neighborhoods, and even cemeteries (8). Extensive observational and experimental evidence has shown that visiting green spaces can improve mood-related outcomes (9–11), whereas living in greener neighborhoods is associated with fewer emotional problems (12), lower cardiovascular disease risk (13), lower incidence of type 2 diabetes (14), and reduced mortality (15, 16).

To explain these health benefits, scholars have proposed multiple theoretical frameworks linking green space to health (17–20). Physical activity is commonly regarded as a key mediator along this pathway. In theory, green spaces provide safer, cleaner, and more comfortable environments that encourage physical activity (18). Considerable evidence has documented a positive association between green space and physical activity (21–23), especially for subjectively perceived green space (24–27). This issue is particularly important today because insufficient physical activity has been identified as one of the major public health challenges of modern society (28), and many countries and regions continue to report levels of physical activity below recommended standards (29, 30). Against this background, physical activity is not only an upstream determinant of health, but also a health-related outcome worthy of attention in its own right. Accordingly, understanding how environmental design can be used to promote physical activity is a meaningful scientific question.

As a dominant structural component of many plant communities, trees play a vital role in both generating visible greenery and shaping spatial structure. A large body of research has documented people’s particular preference for trees within “green” environments (31). This preference may be related to the cues of shelter, safety, and resources historically provided by trees during human evolution (32). For this reason, recent urban greening strategies have increasingly emphasized the presence of trees, especially their visibility. For example, the newly proposed 3–30-300 rule highlights that residents should be able to see at least three trees from their homes (33, 34), and subsequent research has also supported a link between visible trees and health (35).

Despite this progress, several research gaps remain. Specifically, it is reasonable to expect that people may be more willing to engage in physical activity in spaces perceived as “natural,” possibly because of humans’ innate biophilic tendencies or their psychological connection with nature (36–38). Previous work has also suggested that perceived naturalness may shape landscape preference (39). However, what kind of space actually qualifies as “natural” remains unclear. We hypothesized that trees play an important role in shaping people’s perceived naturalness of space, which in turn influences their willingness to be physically active in that space. More specifically, when the visible tree level exceeds a certain threshold, the space may be more likely to be judged as natural and, consequently, more likely to encourage physical activity. To address this question, we designed the present study using photographs taken in urban environments together with quantified visible vegetation elements to examine the relationships among visible tree levels, perceived naturalness, and willingness to engage in physical activity. This work may help answer practical questions in urban greening and urban design regarding not only whether an area is “green enough,” but also which natural elements matter and how much of them is needed. The specific aims of this study were to: (1) examine the associations between visible tree levels, naturalness, and willingness to engage in physical activity; (2) identify the level of visible trees required for a space to be perceived as a “natural space”; and (3) test whether naturalness mediates the relationship between visible tree levels and willingness to engage in physical activity.

## 2 Methods

### 2.1 Study design

With reference to previous environmental psychology studies and considering feasibility, this study adopted an image-based online questionnaire experiment. Participants were asked to provide subjective evaluations of a series of images representing different urban spatial scenes in order to examine their perceptions of naturalness and their willingness to engage in physical activity in those spaces.

This was a within-subject design in which each participant evaluated all images. To reduce order effects, the presentation order of images was fully randomized on the Wenjuanxing platform, meaning that each participant viewed the images in a different sequence.

### 2.2 Study area

The study area was Yucheng District, Ya’an City, Sichuan Province, China. Yucheng District is the political, economic, and cultural center of Ya’an City and is located in the middle reaches of the Qingyi River on the western edge of the Sichuan Basin. It covers approximately 1066.79 km^2^ and is known as the “Rain City” because of its abundant precipitation. In 2022, the permanent resident population of Yucheng District was approximately 369,000, making it the most urbanized and densely populated district in Ya’an.

In the present study, the environmental setting was defined at the district level rather than at the campus level. All image materials were photographed in typical urban streets and open spaces within Yucheng District. The images were not intended to represent campus spaces specifically, nor were they selected according to the formal boundary of Sichuan Agricultural University. Instead, they were selected to capture everyday urban environments in the district where the university is located.

### 2.3 Participants

To ensure that participants had basic familiarity with the local plant types and urban landscape characteristics shown in the images, thereby minimizing possible bias caused by unfamiliarity or exoticness, we recruited university students from Sichuan Agricultural University, which is located in Yucheng District. The recruitment of this student sample was not intended to define the image settings as campus spaces. Rather, students were selected because they study and live in the same district-level urban context from which the images were taken.

Participants were recruited by distributing the online questionnaire link through class WeChat groups and teaching-related group chats managed by instructors, accompanied by a brief description of the research topic and general procedure. Inclusion criteria were: (1) being a currently enrolled student at Sichuan Agricultural University; and (2) having no color blindness or severe visual impairment that would interfere with recognition of image content.

Exclusion criteria included: (1) unusual response patterns (e.g., repeatedly selecting the same response option across many items); (2) abnormally fast completion speed (e.g., an average response time of < 2 s per item); and (3) failure to complete the questionnaire or failure to provide valid informed consent. Before starting the questionnaire, all participants read the study information online and provided electronic informed consent. A total of 337 participants were ultimately included in the analysis. The study was reviewed and supervised by the Ethics Committee of the Southwest University.

### 2.4 Preparation of image stimuli

Photographing points were selected from urban roads, side streets, and open spaces within Yucheng District, Ya’an City. The selection was not restricted to the campus of Sichuan Agricultural University or to spaces immediately adjacent to the university. Rather, the aim was to obtain a heterogeneous set of real-world urban scenes within the same local urban context, with sufficient variation in visible vegetation levels. Image selection and photography followed the principles below:

- Clearly defined spatial boundaries: each image had to contain clear physical boundaries (e.g., buildings, retaining walls, stones, or soil mounds) to form a relatively independent spatial unit that participants could evaluate.
- Presence of basic activity space: each image had to include at least approximately 50 m^2^ of walkable flat area (roughly equivalent to 5 m × 10 m), such as paved ground or lawn, so that participants could plausibly imagine themselves staying or being active in the space.
- Vegetation located within the spatial boundary: all vegetation used for analysis had to be located within the physically bounded space described above, allowing a clear delineation of vegetation “within the space.”

During photography, the following technical requirements were applied:

- The camera was positioned at approximately 170 cm in height to simulate an average adult’s eye level.
- Images were taken from a horizontal forward-facing angle to avoid exaggerated low-angle or high-angle perspectives.
- Whenever possible, photographs were taken under similar weather conditions using natural light, without additional lighting or *post hoc* color enhancement or beautification.
- Recognizable personal faces, license plates, or other privacy-sensitive information were avoided; if unavoidable, the image was blurred or excluded.

**Figure 1:**
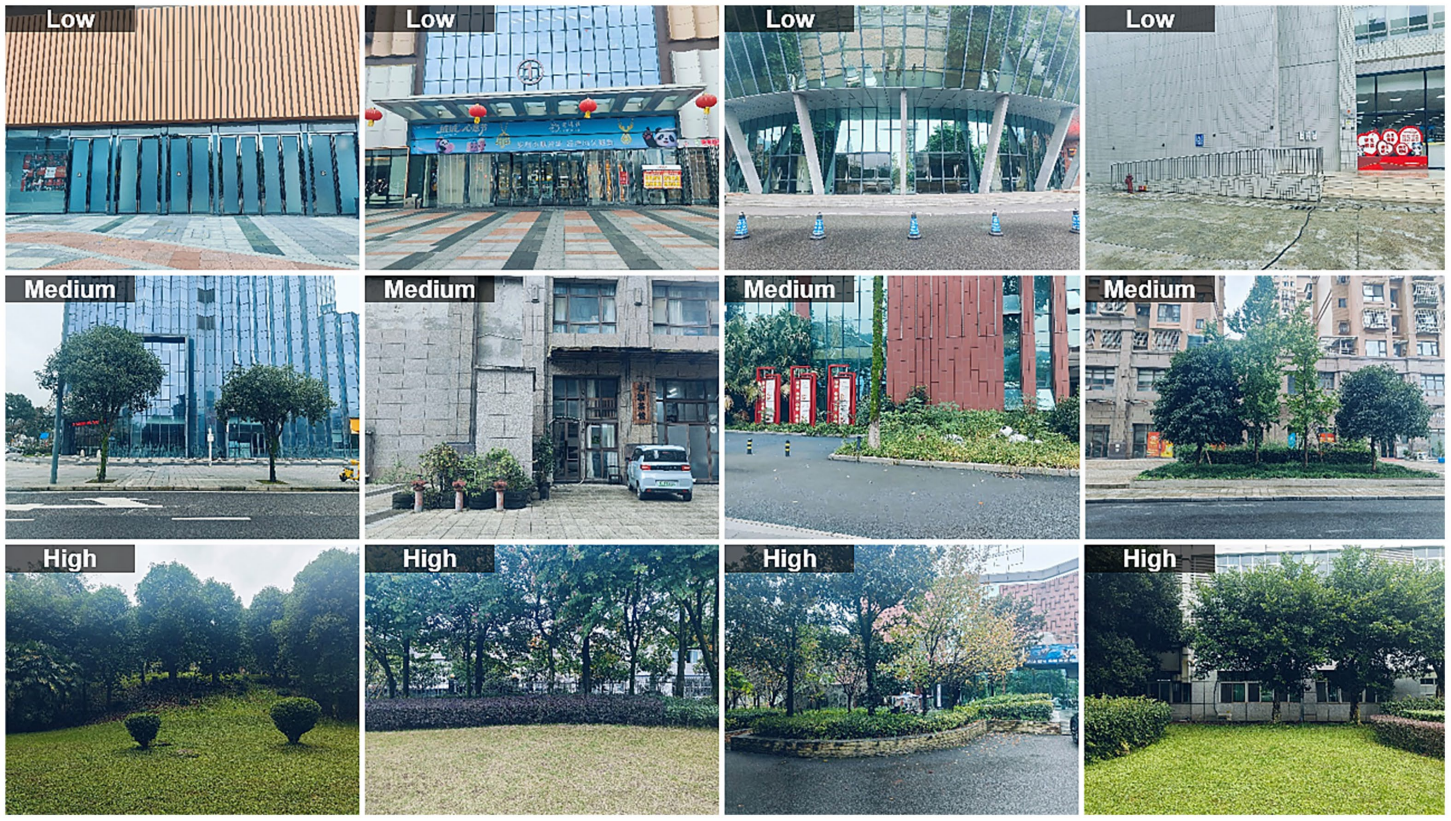
Examples of the experimental stimuli.

To ensure sufficient variation in vegetation levels, we initially selected scenes that broadly represented three categories (Figure 1):

- Spaces with almost no vegetation (low);
- Spaces with a small amount of localized vegetation (medium); and
- Spaces in which vegetation occupied a relatively large proportion of the space (high).

Particular effort was made to ensure that the “partial vegetation” category covered a range from “slightly green” to “substantially vegetated.” After screening, 10 images with almost no vegetation and 30 images containing vegetation were retained, yielding a total of 40 images for questionnaire presentation and subsequent analysis.

### 2.5 Measures

#### 2.5.1 Vegetation indicators

We used semantic segmentation to quantify the visible proportions of vegetation elements in each image (40). Referring to recent image-based urban environment studies using DeepLabV3 + −based semantic segmentation to extract streetscape visual indicators (41), the segmentation procedure in the present study was implemented in Python using MXNet and GluonCV. Specifically, we used the GluonCV deeplab_resnest101_ade model, a DeepLabV3-based semantic segmentation model with a ResNeSt-101 backbone pretrained on the ADE20K dataset. According to the GluonCV Model Zoo, this pretrained model achieved a pixel accuracy of 82.1% and a mean intersection over union of 46.9% on the ADE20K validation set.

Before segmentation, images were resized when necessary to reduce computational burden while preserving the original aspect ratio. The model then generated pixel-level predictions for the 150 ADE20K semantic categories. For each image, the pixel proportion of each category was calculated as the number of pixels assigned to that category divided by the total number of pixels in the processed image.

The vegetation variables were operationally defined according to the ADE20K SceneParse150 visual-semantic label system rather than strict botanical taxonomy. According to the ADE20K label file, vegetation-related labels include “tree,” “grass,” “plant, flora, plant life,” “flower,” and “palm, palm tree.” In the present study, we extracted three types—Tree, Grass, and Other plant (corresponding to “plant, flora, plant life” label)—because these categories were the vegetation-related semantic labels most consistently represented in the selected urban scenes and most relevant to our research aims.

Specifically, Tree referred to pixels classified as the ADE20K “tree” label, Grass referred to pixels classified as the ADE20K “grass” label, and Other plant referred to pixels classified as the ADE20K “plant, flora, plant life” label. These labels were mutually exclusive at the pixel level because each pixel received only one predicted ADE20K class. Therefore, Other plant did not include pixels classified as Tree or Grass and should not be interpreted as total plant cover or total vegetation. Shrubs, hedges, herbaceous vegetation, and mixed vegetation forms were not manually reassigned according to botanical criteria; rather, they were retained according to the semantic label predicted by the model. Throughout the manuscript, these variables are therefore interpreted as model-derived visible semantic categories, not as precise botanical or ecological measurements.

To facilitate quality control, the predicted segmentation masks were exported as color-coded overlay images superimposed on the original photographs. These overlay images were visually inspected by the research team to identify obvious segmentation failures, such as large non-vegetation areas being misclassified as vegetation or clearly visible vegetation being omitted by the model. The final Tree, Grass, and Other plant proportions were therefore treated as model-derived estimates of visible vegetation composition.

#### 2.5.2 Naturalness

Participants rated the perceived naturalness of each image using a 4-point Likert scale (1 = very unnatural; 2 = somewhat unnatural; 3 = somewhat natural; 4 = very natural). To avoid an ambiguous middle option, no neutral category was provided, thereby encouraging participants to make a directional judgment toward either the natural or the unnatural side. This approach was informed by the design and refinement strategies of other measurement instruments (42).

In the statistical analyses:

Scores from 1 to 4 were treated as a continuous variable representing the level of perceived naturalness;

The responses were also recoded into a binary variable for classification analyses:

Ratings of 1 or 2 were coded as 0 (“unnatural”);

Ratings of 3 or 4 were coded as 1 (“natural”).

#### 2.5.3 Physical activity willingness

Participants used an 11-point numerical rating scale (NRS) to indicate their willingness to engage in physical activity such as walking or group exercise in the space depicted in the image (0 = completely unwilling; 10 = very willing). This score was treated as a continuous variable representing subjective willingness to engage in physical activity in that space (43).

#### 2.5.4 Covariates

To control for the influence of physical space characteristics on subjective evaluations, we included two covariates subjectively rated by the researchers:

Estimated space area (m^2^): estimated according to the relative scale of the walkable area shown in the image;

Walkability rating (1–10): independently judged by two researchers based on ground flatness, the number of obstacles, ground material, and related characteristics, with higher scores indicating greater suitability for walking and light physical activity.

After independently scoring the images, the two raters discussed cases with large discrepancies until consensus was reached.

### 2.6 Statistical analysis

#### 2.6.1 Correlation analysis

Because each image was rated by multiple participants, individual ratings were not statistically independent. To reduce the dependence introduced by repeated ratings, we first aggregated the data at the image level by calculating the mean naturalness score, mean physical activity willingness score, and other subjective ratings across all participants for each image. Pearson correlation analyses were then performed at the image level to examine the linear associations between vegetation indicators and both perceived naturalness and willingness to engage in physical activity.

#### 2.6.2 Regression analysis: generalized estimating equations (GEE)

Before the regression analyses, we used variance inflation factors (VIFs) to examine multicollinearity among predictors and confirmed that all VIF values were below the commonly used threshold of 5.0 (44). Given the cross-nested participant-by-image structure of the raw data, we used generalized estimating equations (GEE) to more rigorously test the relationships between vegetation and naturalness/physical activity willingness. In the models, the independent variables were the three vegetation-related pixel proportion indicators—Tree, Grass, and Other plant—entered separately into the models; the covariates were estimated space area and walkability rating; and participants were specified as the subject variable, with image ID treated as the repeated-measures clustering unit to account for the clustering created by repeated ratings of the same image by multiple participants. An unstructured covariance matrix (UN) was used.

For the continuous outcomes—naturalness ratings (1–4 points, treated continuously) and physical activity willingness (0–10 points)—we used models assuming a normal distribution with an identity link. Because both outcomes showed only small absolute skewness values (naturalness skewness = 0.03; physical activity willingness skewness = 0.13) (45), the normality assumption was considered acceptable and no transformation was applied. For the recoded binary naturalness outcome (0 = unnatural, 1 = natural), we fitted a binary logistic model with a logit link and reported odds ratios (ORs) and 95% confidence intervals for the vegetation indicators.

#### 2.6.3 Exploratory cut-off analysis

To explore whether visible vegetation proportions could distinguish images perceived as natural from those perceived as unnatural, we conducted receiver operating characteristic (ROC) analyses at the image level. The ROC analysis was based on 40 images rather than individual participant ratings.

First, each participant’s naturalness rating was recoded into a binary response: ratings of 1 or 2 were coded as 0 (“unnatural”), whereas ratings of 3 or 4 were coded as 1 (“natural”). Second, for each image, we calculated the proportion of participants who classified that image as natural. Third, images were assigned an image-level binary naturalness label: images classified as natural by at least 50% of participants were coded as 1 (“natural space”), whereas the remaining images were coded as 0 (“unnatural space”).

Using this image-level binary naturalness label as the outcome, ROC curves were plotted separately for Tree, Grass, and Other plant pixel proportions. The area under the curve (AUC) was used to evaluate the discriminative ability of each vegetation indicator. An AUC ≥ 0.70 was interpreted as fair discrimination and an AUC ≥ 0.80 as good discrimination Çorbacıoğlu Ş and Aksel (46). When the AUC suggested acceptable discrimination, the optimal cut-off was estimated using the Youden index, calculated as Sensitivity + Specificity – 1 (47). The resulting cut-off was interpreted as an exploratory image-level estimate within the present sample of 40 images, rather than as a definitive or generalizable threshold.

#### 2.6.4 Mediation analysis

Structural equation modeling (SEM) was used to examine the pathways and mediation effects among the variables of interest. Three simple mediation models were specified separately for the three vegetation types. In these models, the 1–4 naturalness rating served as the mediator, and physical activity willingness was specified as the dependent variable. Maximum likelihood (ML) estimation was used. For all paths, standard errors and confidence intervals were generated using bias-corrected bootstrapping with 10,000 resamples (48), a procedure that is robust to non-normality (49–51). An indirect effect that differed significantly from zero (i.e., the product of the constituent path coefficients) was taken as evidence of mediation (52, 53). Consistent with the regression analyses above, walkability and estimated space area were included as covariates. Because each simple mediation model was saturated, and remained saturated after the covariates were added, overall model-fit indices were not reported. To avoid multicollinearity problems in SEM, we again examined VIFs and did not observe serious multicollinearity (the highest VIF was 4.5 for naturalness). A *p*-value < 0.05 was considered statistically significant.

#### 2.6.5 Sensitivity analysis

Given that the original dataset had a crossed structure, with participant ratings nested within both images and participants, we conducted an additional sensitivity analysis to provide a robustness check for the participant-level GEE analyses. Specifically, image-level aggregated values were used to run linear regression and logistic regression models to examine the associations between the three vegetation variables and naturalness ratings, expressed in both continuous and binary forms, as well as willingness to engage in physical activity. Consistent with the main analyses, walkability and estimated area were included as covariates.

## 3 Results

### 3.1 Participant characteristics

A total of 337 university students were included. The sample was relatively concentrated in age, with a mean age of approximately 19.9 years (Table 1). The sex distribution was broadly balanced, with males accounting for about 48% and females about 52%.

**Table 1:** Basic characteristics of the participants (*N* = 337).

**Variable**	**Category**	**Mean (SD)**	**Median (IQR)**	**Range**	**n (%)**
Age (years)	–	19.88 (1.29)	20.00 (2.00)	18.00–27.00	–
Sex	Male	–	–	–	162 (48.1)
	Female	–	–	–	175 (51.9)

### 3.2 Image-level correlations

The correlation results based on image-level means (or proportions) are shown in Table 2. Overall, perceived naturalness and willingness to engage in physical activity were very strongly positively correlated (*r* ≈ 0.95, *p* < 0.001), indicating that participants were more willing to be physically active in spaces they perceived as more natural. The three vegetation-related pixel indicators (Tree, Grass, and Other plant) were also all significantly positively correlated with perceived naturalness (continuous scores) and physical activity willingness. In particular, the correlations between Tree pixel proportion and both naturalness and physical activity willingness ranged from 0.84 to 0.90, suggesting that visible tree cover may be more influential than Grass or Other plant pixels in shaping the subjective impression of a “natural space.”

**Table 2:** Correlation coefficients among image-level variables (based on means/proportions, *N* = 40 images).

**Variable**	**1**	**2**	**3**	**4**	**5**
1 Naturalness rating	1	-	-	-	-
2 Physical activity willingness	0.949**	1	-	-	-
3 Tree	0.841**	0.900**	1	-	-
4 Grass	0.572**	0.560**	0.465**	1	-
5 Other Plant	0.478**	0.481**	0.313*	−0.030	1

Correlation coefficients were calculated from the mean value or proportion of each variable across all participants for each image. Significance: *p < 0.05, **p < 0.01.

### 3.3 Generalized estimating equations (GEE)

#### 3.3.1 Vegetation and perceived naturalness (continuous)

In the GEE analyses, the pixel proportions of Tree, Grass, and Other plant were all significantly associated with higher naturalness ratings (1–4 points) (Table 3). Taking Tree as an example, its regression coefficient was 0.631 (95% CI: 0.554 to 0.707, *p* < 0.001), indicating that, with other variables held constant, a one-unit increase in Tree pixel proportion (i.e., from 0 to 100%) was associated with an average increase of approximately 0.631 points in the naturalness rating.

**Table 3:** GEE results for the associations of vegetation pixel proportions with perceived naturalness and physical activity willingness.

**Outcome variable**	**Predictor**	**B / OR**	**95% CI Lower**	**95% CI Upper**	***p*-value**
Naturalness rating	Tree	0.631	0.554	0.707	<0.001
	Grass	0.449	0.292	0.607	<0.001
	Other plant	0.566	0.391	0.741	<0.001
Naturalness rating (binary, natural = 1)	Tree	5.368	4.017	7.174	<0.001
	Grass	13.738	8.372	22.543	<0.001
	Other plant	18.083	8.729	37.459	<0.001
Physical activity willingness	Tree	1.915	1.639	2.191	<0.001
	Grass	0.501	−0.121	1.123	0.114
	Other plant	0.997	0.507	1.488	<0.001

For the continuous outcomes (naturalness rating and physical activity willingness), the table reports regression coefficients (B). For the binary naturalness outcome, the table reports odds ratios (OR). All models were adjusted for estimated walkable area and walkability rating.

#### 3.3.2 Vegetation and binary naturalness ratings

In the logistic model using whether a participant rating was classified as natural (0 = unnatural, 1 = natural) as the outcome, the odds ratios (ORs) for all three vegetation-related indicators were significantly greater than 1 (Table 3). This suggests that higher Tree, Grass, and Other plant pixel proportions increased the likelihood that a participant rating would fall into the natural category. The OR for Other plant was particularly large (OR = 18.083), indicating that the proportion of pixels assigned to the ADE20K Other plant label showed a strong association with binary naturalness ratings.

#### 3.3.3 Vegetation and willingness to engage in physical activity

In the GEE model using physical activity willingness (0–10 points) as the outcome, Tree and Other plant pixel proportions were significantly and positively associated with willingness to engage in physical activity (Table 3). Specifically, Tree showed the strongest association (*B* = 1.915, 95% CI: 1.639 to 2.191, *p* < 0.001), followed by Other plant (*B* = 0.997, 95% CI: 0.507 to 1.488, *p* < 0.001). In contrast, the association between Grass and physical activity willingness was not statistically significant (*B* = 0.501, 95% CI: −0.121 to 1.123, *p* = 0.114). These results indicate that visible trees, rather than grass, showed the most robust association with participants’ willingness to be physically active in the depicted spaces.

### 3.4 Vegetation thresholds: exploratory ROC analysis

The image-level ROC analysis showed that Tree pixel proportion had good ability to distinguish images perceived as natural spaces from those perceived as unnatural spaces (AUC = 0.90) (Table 4). Based on the Youden index, the exploratory optimal cut-off for Tree pixel proportion was 0.277, with sensitivity = 1.000, specificity = 0.733, and Youden index = 0.733. This suggests that, within the present set of 40 images, images with Tree pixel proportions above approximately 27.7% were more likely to be classified as natural spaces by the majority of participants. However, because the ROC analysis was based on a limited number of images, this value should be interpreted as a preliminary cut-off estimate rather than a definitive or generalizable threshold.

**Table 4:** Receiver operating characteristic analysis for vegetation indicators in identifying images perceived as natural spaces.

**Test variable**	**AUC**	**SE**	**p value**	**95% CI**
Tree	0.909	0.055	<0.001	0.801 to 1.000
Grass	0.772	0.083	0.004	0.609 to 0.935
Other plant	0.700	0.091	0.036	0.522 to 0.878

AUC = area under the receiver operating characteristic curve; SE = standard error; CI = confidence interval. Standard errors and confidence intervals were estimated under the nonparametric assumption. The null hypothesis was AUC = 0.5. At least one tie was observed between the positive and negative actual-state groups for Tree, Grass, and Other plant; therefore, the statistics may be biased.

The AUC values for Grass and Other plant were lower than that for Tree, indicating weaker discriminative ability. Therefore, the subsequent cut-off interpretation focused primarily on Tree. Overall, the ROC results support the prominent role of visible trees in shaping natural-space perception, but the estimated cut-off requires validation in larger and more diverse image datasets.

### 3.5 Mediation analysis

**Figure 2:**
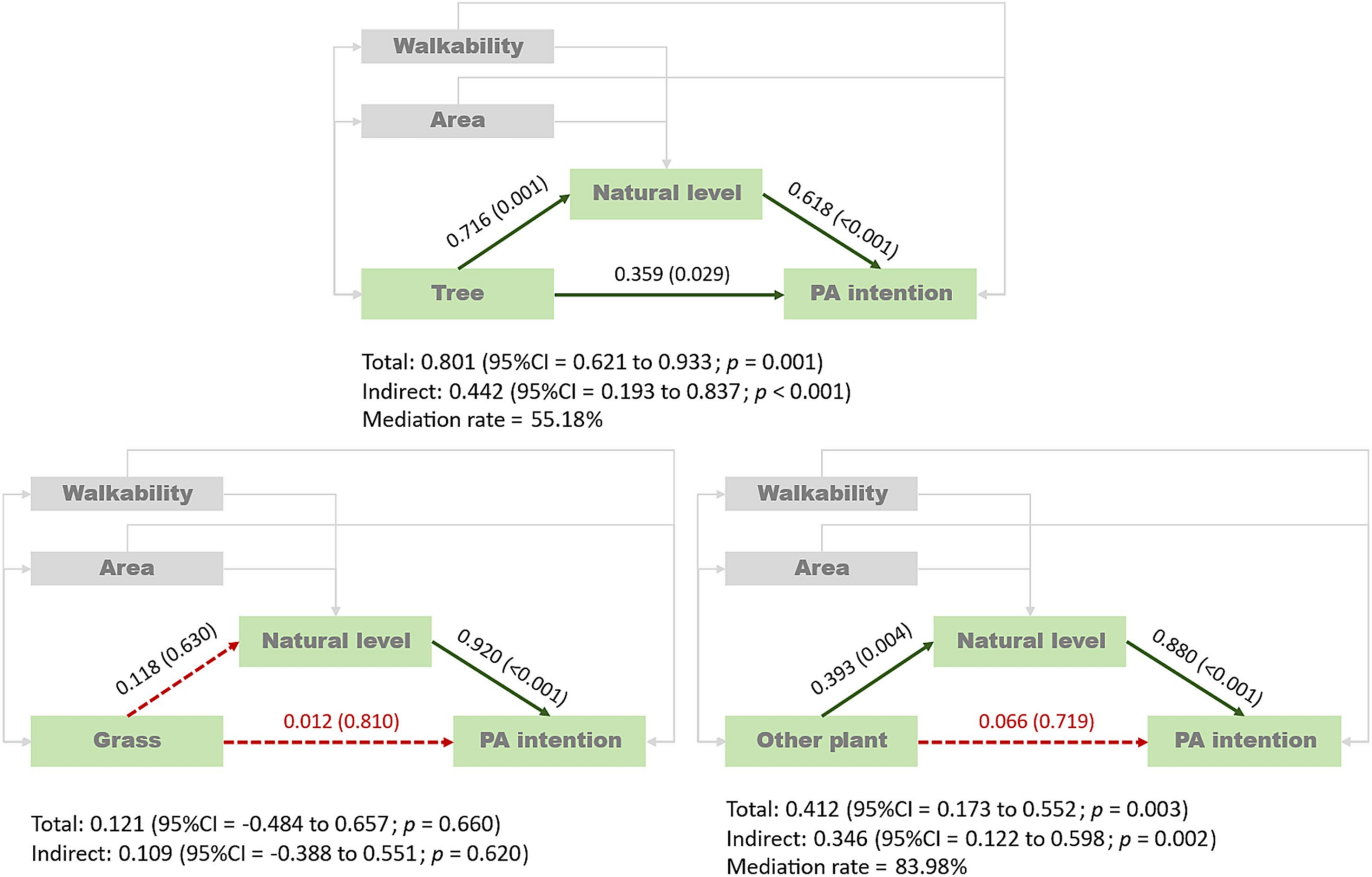
Mediation analysis based on image-level data. Gray boxes and arrows represent covariates and control paths; green solid lines indicate significant paths, whereas red dashed lines indicate non-significant paths. The numbers outside the parentheses are standardized regression coefficients, and the numbers inside the parentheses are *p*-values.

The mediation analyses showed that naturalness was a significant mediator in the associations of Tree and Other plant with willingness to engage in physical activity (Figure 2). Notably, naturalness fully mediated the associations of Other plant with physical activity willingness, as reflected by strong total associations but non-significant direct associations. In terms of mediation magnitude, naturalness explained 55.18% and 83.98% of the total associations.

### 3.6 Sensitivity analysis

In the image-level sensitivity analyses, the main findings were generally consistent with the participant-level analyses (Tables 5, 6). Tree proportion was positively associated with naturalness rating (*B* = 1.827, 95% CI: 1.328 to 2.327, *p* < 0.001), physical activity willingness (*B* = 3.954, 95% CI: 3.168 to 4.740, *p* < 0.001), and binary naturalness classification (OR = 325,705.837, 95% CI: 18.950 to 5.598 × 10^9^, *p* = 0.011). Other plant proportion was also positively associated with naturalness rating (*B* = 3.490, 95% CI: 1.410 to 5.569, *p* = 0.002), physical activity willingness (*B* = 7.073, 95% CI: 3.064 to 11.081, *p* = 0.001), and binary naturalness classification (OR = 735,349,830.800, 95% CI: 1.069 to 5.060 × 10^17^, *p* = 0.049). These unusually large OR values indicate that, at the image level, the vegetation-related indicators in images classified as natural were almost completely higher than those in images classified as unnatural. In contrast, Grass proportion was not significantly associated with any outcome in the image-level models. Given the small number of images and the very large ORs with wide confidence intervals, the logistic regression results should be interpreted cautiously. Overall, these sensitivity analyses support the robustness of the main findings, particularly the prominent role of visible trees.

**Table 5:** Image-level sensitivity analyses for continuous outcomes.

**Outcome**	**Vegetation predictor**	**B (95% CI)**	**p**
Naturalness rating	Tree	1.827 (1.328 to 2.327)	<0.001
Naturalness rating	Grass	0.593 (−1.594 to 2.779)	0.586
Naturalness rating	Other plant	3.490 (1.410 to 5.569)	0.002
Physical activity willingness	Tree	3.954 (3.168 to 4.740)	<0.001
Physical activity willingness	Grass	1.173 (−3.096 to 5.441)	0.581
Physical activity willingness	Other plant	7.073 (3.064 to 11.081)	0.001

Analyses were conducted at the image level using aggregated image-level values. Each model included one vegetation predictor and was adjusted for walkability and estimated area. B represents the unstandardized regression coefficient.

**Table 6:** Image-level sensitivity analyses for binary naturalness ratings.

**Outcome**	**Vegetation predictor**	**OR (95% CI)**	**p**
Binary naturalness rating	Tree	325,705.837 (18.950 to 5.598 × 10^9^)	0.011
Binary naturalness rating	Grass	1.788 × 10^14^ (6.630 × 10^−10^ to 4.820 × 10^37^)	0.233
Binary naturalness rating	Other Plant	735,349,830.800 (1.069 to 5.060 × 10^17^)	0.049

Analyses were conducted at the image level using aggregated image-level values. Each model included one vegetation predictor and was adjusted for walkability and estimated area. OR represents Exp(B). Because the logistic regression models were based on only 40 images and produced very large ORs with wide confidence intervals, these results should be interpreted cautiously.

## 4 Discussion

### 4.1 General discussion

Natural environments are important settings for physical activity, and the presence of trees may play a particularly meaningful role in shaping both perceived naturalness and willingness to engage in physical activity. In this study, we conducted an image-based experiment among university students and used semantic segmentation to examine the relationships between visible tree levels, naturalness ratings, and physical activity willingness, as well as the mediating role of naturalness in the association between visible tree levels and physical activity willingness. In addition, after dichotomizing the naturalness ratings, we explored the critical visible-tree level at which participants began to classify a space as natural rather than unnatural. Given the available ADE20K vegetation-related labels, we also included Grass and Other plant categories in the analyses.

Our analyses showed that visible vegetation was generally positively associated with perceived naturalness and willingness to engage in physical activity. However, the strength and robustness of these associations differed across vegetation-related semantic categories. Tree proportion showed the most consistent pattern across the image-level correlations, participant-level GEE models, ROC analysis, and image-level sensitivity analyses. Other plant proportion also showed positive associations with naturalness and physical activity willingness, whereas the associations involving Grass were weaker and less stable, especially in the image-level sensitivity analyses.

After recoding participants’ ratings into a binary natural-versus-unnatural classification, all three vegetation-related indicators produced very large OR values in GEE and particularly in the image-level logistic regression sensitivity analysis. Inspection of the data suggested that this pattern arose because, in images classified as natural by most participants, the detected levels of all three vegetation-related semantic categories were generally much higher than in the reference group of images classified as unnatural by most participants (54). This further underscores the importance of visible vegetation for the experience of spatial naturalness.

The GEE results and the image-level sensitivity analyses consistently indicated that Tree was the most robust vegetation indicator associated with physical activity willingness. Prior to our study, researchers had already explored the relationship between tree density and spatial preference and found that the positive association between tree density and preference gradually attenuates as density increases, suggesting diminishing returns from adding more trees (55). Unlike that work, we preliminarily focused on the threshold level of vegetation at which people begin to perceive a space as natural. In the exploratory cut-off analysis, only the model based on Tree showed sufficiently strong classification performance (AUC = 0.90, with the lower bound of the 95% confidence interval exceeding 0.70), so only this indicator was examined further. The resulting cut-off was 0.277 (sensitivity = 1.000; specificity = 0.733; Youden index = 0.733). In other words, when the Tree pixel proportion exceeded 27.7% of the image, the depicted space was more likely to be judged as a “natural space.” This finding is similar to earlier street-view image research (56), which showed that perceived naturalness and safety increase as tree density rises from “no trees” to “a few trees” and then to “many trees.” Together, these findings highlight that trees—especially trees that are visible from specific positions and viewing angles—are central to how people experience nature. This also suggests that, for green spaces, the “dose” of trees matters: both the quality and the quantity of greenery may determine whether a space can meaningfully influence residents’ experiences and health. In this sense, our results align well with the “3 visible trees” component of the 3–30-300 rule (57) and provide useful guidance for future urban greening practice. Meanwhile, it should be noted that although the ROC analysis suggested an exploratory Tree pixel proportion cut-off of 27.7%, this value should be interpreted cautiously. The ROC analysis was conducted at the image level and was based on only 40 images. Therefore, the estimate may be sensitive to the specific image set, the distribution of tree cover across images, the binary coding rule used to define natural spaces, and possible segmentation errors.

In the exploratory mediation analyses based on image-level values, the associations of Tree and Other plant with physical activity willingness were significantly mediated by naturalness. Given the limited number of image-level vegetation values, these results should be interpreted as highly exploratory. Nevertheless, they suggest that perceived naturalness may be an important perceptual pathway linking visible tree to willingness to engage in physical activity. Notably, for Other plant, the direct effects became non-significant after naturalness was introduced into the model despite significant total effects, indicating full mediation (58). Although other positive pathways may also exist, this result emphasizes the potential role of perceived naturalness in the process through which natural elements stimulate willingness to engage in physical activity. Prior to our study, Liu et al. (59) found that perceived naturalness of campus green spaces predicted green-space use among university students. Given that physical activity, such as walking, is one of the most common ways in which green spaces are used, our findings provide partial support for Liu et al. (59) by further suggesting that greenery needs to be perceived and experienced as natural, rather than merely being physically present.

From an applied perspective, the present findings should be interpreted as evidence concerning environmental perception and activity tendency rather than actual physical activity behavior. The results suggest that visible vegetation, particularly visible trees, may contribute to stronger perceived naturalness and greater stated willingness to be active in urban spaces. However, this does not mean that increasing visible tree cover would necessarily increase real-world physical activity levels. Actual behavior is influenced by many additional factors, including accessibility, safety, social context, weather, time availability, personal preference, and existing activity habits. Therefore, the public health implications of this study should be viewed as preliminary. The findings may help inform the perceptual and visual design of urban spaces, but future field studies are needed to test whether such visual-perceptual advantages translate into actual space use, walking volume, duration of stay, or exercise intensity.

### 4.2 Limitations

In prior experiments using image- or video-based stimuli, balancing ecological validity and internal validity has always been challenging. Researchers have either used fully realistic images such as those in the present study (55, 60, 61), edited real images using post-processing software to improve comparability (56, 62, 63), or relied entirely on synthetic scenes (43, 64, 65). More realistic images can produce impressions that are closer to the real world, but real-world scenes inevitably differ in many ways beyond the focal variables of interest. To preserve ecological validity, we used real urban scenes and tested local students who were familiar with the environmental features shown. Although we initially considered editing the images to increase comparability, the final experiment used a randomized crossover exposure design in which participants viewed all images; any obvious manual modifications to similar scenes could have been noticed by participants and may have influenced their judgments. We therefore retained the original photographs. Although we attempted to ensure approximate similarity in space size and adjusted for walkability and estimated area in the analyses, we could not fully standardize built features such as buildings. In addition, we could not ensure that the plant species and structural forms were equivalent across scenes, and different tree forms or species may influence human perceptions differently (66, 67). Accordingly, substantial confounding remains possible, and the findings should be interpreted with caution.

Another limitation concerns the relationship between the participant sample and the environmental settings. Although participants were recruited from Sichuan Agricultural University, the images were selected to represent general urban streets and open spaces in Yucheng District rather than campus spaces specifically. Recruiting local university students helped ensure basic familiarity with the local vegetation and urban landscape features, but it also limits the generalizability of the findings. The results should therefore be interpreted as reflecting the perceptions of a relatively homogeneous student sample toward district-level urban environments in Ya’an. Future studies should recruit more diverse local residents and include images from multiple urban districts or cities to test whether the observed associations between visible vegetation, perceived naturalness, and physical activity willingness are robust across broader populations and environmental contexts.

Because the study used a fully randomized crossover exposure design, and to reduce participant fatigue, we strictly limited the experimental materials to 40 images. This meant that the actual range of values for the independent variables, such as tree level, was relatively limited. As a result, the statistical power of some analyses, such as the mediation models and ROC analysis, may have been reduced. In addition, because of the nested data issue inherent in the crossover design, the cut-off analyses were based only on average scores or classifications across 40 images, which further limits the robustness of the threshold estimates. Overall, we emphasize that the analyses conducted here are exploratory in nature and should be extended and validated in future studies with more comprehensive designs and resources.

Our outcome variable reflected willingness to engage in physical activity after image exposure rather than actual physical activity behavior in real environments. Although behavioral intention is theoretically an important antecedent of behavior, the two are not equivalent. A participant’s stated willingness to be active while viewing static images cannot fully represent actual entry into the space, time spent there, walking levels, or other objective physical activity behavior in real settings. Moreover, static images cannot fully capture dynamic environmental cues such as sound, temperature, air movement, spatial depth, and traces of social activity. Therefore, the findings should be understood as preliminary evidence regarding environmental perception and activity tendency rather than direct proof of behavioral responses in the real world.

The key exposure indicators in this study may be subject to measurement error and should be interpreted as model-derived visual-semantic estimates rather than precise botanical or ecological measurements. Although the use of a DeepLabV3-based model pretrained on ADE20K provided an efficient and reproducible way to quantify visible vegetation elements, the reported benchmark performance of this model was based on the ADE20K validation set and does not directly represent segmentation accuracy for the specific images used in the present study. In our analysis, we visually inspected the segmentation overlays to identify obvious segmentation failures, but we did not conduct formal pixel-level manual annotation validation or inter-rater consistency analysis. Therefore, the Tree, Grass, and Other plant proportions should be interpreted as estimated visible semantic categories rather than exact vegetation areas.

In addition, although we standardized the operational definitions of Tree, Grass, and Other plant according to the ADE20K label system, these categories do not represent strict botanical classifications. ADE20K provides semantic category labels, such as “tree,” “grass,” and “plant, flora, plant life,” but does not offer detailed botanical rules for distinguishing tree canopy, shrubs, hedges, herbaceous vegetation, and mixed vegetation forms in complex urban scenes. Thus, shrubs or hedge-like vegetation were not manually assigned to Tree by the researchers; rather, they were classified according to the label predicted by the model. Segmentation errors and label ambiguity may have affected the subsequent correlation, regression, ROC, and mediation analyses. Future studies should combine automated semantic segmentation with manual point-sampling validation, pixel-level annotation, inter-rater validation, or vegetation-specific segmentation models to improve measurement precision and distinguish tree canopy, shrubs, hedges, grass, herbaceous vegetation, and other vegetation more accurately.

## 5 Conclusion

Using photographs of real urban scenes, this study preliminarily examined the relationships between visible vegetation levels—especially visible tree levels—perceived naturalness, and willingness to engage in physical activity. The results showed that visible tree proportion had the most consistent and robust associations with perceived naturalness, natural-space classification, and willingness to engage in physical activity. Pixels assigned to the ADE20K Other plant label also showed positive associations, whereas Grass showed weaker and less stable associations across analyses. Further analyses indicated that when the visible tree area accounted for approximately 27.7% of the image, the space was more likely to be judged as a “natural space” by the majority of participants. Overall, this study provides preliminary evidence for understanding what kind of visible greenery may be associated with stronger perceived naturalness and greater willingness to engage in physical activity in image-based urban scenes. Compared with simply considering whether greening is present, urban environmental design and greening practice may benefit from paying greater attention to the visibility of natural elements such as trees and to the subjective experience of naturalness they generate. However, because this study used static image stimuli and measured willingness rather than actual physical activity behavior, the findings should be limited to environmental perception and activity tendency. Future research should examine whether these perceptual responses translate into real-world space use, walking volume, duration of stay, or exercise intensity.

## Data Availability

The raw data supporting the conclusions of this article will be made available by the authors, without undue reservation.
